# Dysregulation of glucose homeostasis in nicotinamide nucleotide transhydrogenase knockout mice is independent of uncoupling protein 2

**DOI:** 10.1016/j.bbabio.2009.06.005

**Published:** 2009-12

**Authors:** Nadeene Parker, Antonio J. Vidal-Puig, Vian Azzu, Martin D. Brand

**Affiliations:** a MRC Mitochondrial Biology Unit, Hills Road, Cambridge CB2 0XY, UK; b Metabolic Research Laboratories, Level 4, Institute of Metabolic Science, Box 289, Addenbrooke's Hospital, Cambridge CB2 0QQ, UK

**Keywords:** CLAMS, Comprehensive Laboratory Animal Monitoring System, GSIS, glucose stimulated insulin secretion, KO, knockout, NNT, nicotinamide nucleotide transhydrogenase, ROS, reactive oxygen species, TPMP^+^, Triphenylmethylphosphonium, UCP, uncoupling protein, WT, wild-type, Uncoupling protein 2 (UCP2), Nicotinamide nucleotide transhydrogenase (NNT), Proton leak, Glucose tolerance, Glucose stimulated insulin secretion (GSIS)

## Abstract

Glucose intolerance in C57Bl/6 mice has been associated with mutations in the nicotinamide nucleotide transhydrogenase (*Nnt*) gene. It has been proposed that the absence of NNT from mitochondria leads to increased mitochondrial reactive oxygen species production and subsequent activation of uncoupling protein 2 (UCP2). Activation of UCP2 has been suggested to uncouple electron transport from ATP synthesis in pancreatic beta cell mitochondria thereby decreasing glucose tolerance due to decreased insulin secretion through lower ATP/ADP ratios. The hypothesis tested in this paper is that UCP2 function is required for the dysregulation of glucose homeostasis observed in NNT ablated mice. Single and double *Nnt* and *Ucp2* knockout mouse lines were used to measure glucose tolerance, whole animal energy balance and biochemical characteristics of mitochondrial uncoupling. As expected, glucose tolerance was diminished in mice lacking NNT. This was independent of UCP2 as it was observed either in the presence or absence of UCP2. The range of metabolic parameters examined in the mice and the proton conductance of isolated mitochondria remained unaltered in this double NNT and UCP2 knockout model. Ablation of UCP2 did not itself affect glucose tolerance and therefore previous observations of increased glucose tolerance of mice lacking UCP2 were not confirmed. We conclude that the decreased glucose tolerance in *Nnt* knockout mice observed in our experiments does not require UCP2.

## 1 Introduction

Uncoupling protein 2 (UCP2) is a member of a family of mitochondrial inner membrane uncoupling proteins that also includes UCP1, UCP3 and the adenine nucleotide translocase. All these proteins are proposed to share an ancestral function similar to that of UCP1 which uncouples ATP synthesis from substrate oxidation in brown fat by catalysing a leak of pumped protons back into the mitochondrial matrix. The resultant dissipation of the mitochondrial protonmotive force produces heat at the expense of ATP production [1,2]. Unlike UCP1, the substantially lower levels of the other uncoupling proteins mean it is unlikely that they produce a physiologically significant amount of heat [3]. In mitochondria isolated from a variety of tissues and cell lines, uncoupling protein activity is increased following exposure to fatty acids [4–7], superoxide [8], reactive oxygen species (ROS) derivatives such as hydroxynonenal, and other reactive alkenals [9,10]. This uncoupling activity can be inhibited by purine nucleoside di- or triphosphates [7,11], carboxyatractylate or bongkrekate [11,12]. The uncoupling activity of UCP1 has long been identified as the cause of heat production in the brown fat of neonates and small mammals [2]. The physiological function of other proteins in the family, however, remains controversial — proposals include protection against mitochondrial ROS production [13–16], export of fatty acids from the mitochondrial matrix [17–19], and regulation of insulin secretion in pancreatic beta cells [20–27].

In pancreatic beta cells, the release of insulin is tightly coupled to the cellular ATP/ADP ratio. There is growing evidence that UCP2 is a negative regulator of glucose stimulated insulin secretion (GSIS) [20–27]. The most compelling observations are that GSIS is improved in *Ucp2* ablated mice [21] or following UCP2 knockdown in the pancreatic beta cell model, INS-1E [27]. Speculation has therefore grown as to the physiological function of UCP2 in the pancreatic beta cell [28]. One theory is that the presence of UCP2 in mitochondria is required to limit damaging ROS production and the subsequent beta cell dysfunction that would arise. This is supported by data showing higher mitochondrial membrane potential and elevated ROS levels in isolated pancreatic beta cells from *Ucp2* ablated mice compared to WT controls [24]. However, the elevation of ROS in UCP2-ablated pancreatic beta cells did not affect improvement in glucose homeostasis over the long term and did not lead to loss of pancreatic beta cell mass [24]. Other theories suggest that UCP2 is directly involved in the physiological attenuation of GSIS by other effectors such as fatty acids [29].

**Fig. 1:**
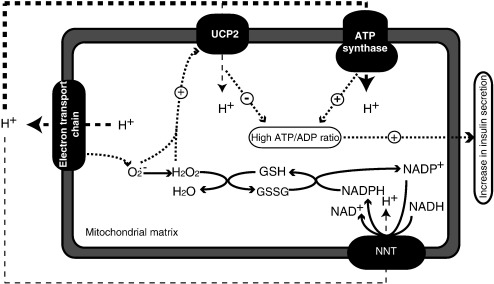
Hypothesised mechanism of dysregulation of glucose homeostasis by NNT and UCP2. Detoxification of superoxide (O_2_^•−^) produced by the electron transport chain in the mitochondrial matrix is dependent upon the reduced glutathione pool (GSH). Re-reduction of glutathione disulfide (GSSG) is in turn dependent on the reduced NADPH. Evidence suggests that NNT is involved in the detoxification of O_2_^•−^ via the maintenance of a highly reduced glutathione pool through the NADPH-dependent glutathione reductase. Inactivation of NNT is proposed to cause build up of harmful O_2_^•−^, activation of UCP2 and dissipation of the protonmotive force generated by the electron transport chain to drive the F_1_F_o_-ATP synthase. Activation of a proton leak through UCP2 has been shown to decrease the cellular ATP/ADP ratios needed to drive insulin secretion.

Nicotinamide nucleotide transhydrogenase (NNT) is a ubiquitous mitochondrial inner membrane protein. When the mitochondrial membrane potential is high, NNT couples proton translocation to a redox reaction reducing mitochondrial NADP^+^ while oxidising NADH [30–32]. NNT ablation has been suggested to be the main cause of the glucose intolerant phenotype of certain C57Bl/6J strain mice, which carry a mutation in the *Nnt* gene [33–35]. In support of this position, ablation of *Nnt* in a non-C57Bl/6J background also results in mice that are glucose intolerant and have decreased plasma insulin [33]. Pancreatic islets from *Nnt* knockout (*Nnt*KO) mice have a normal response to the potassium channel blocker tolbutamide, implying that the regulation of insulin secretion downstream of the K_ATP_ channel is normal. Their low beta cell ATP content following stimulation with 20 mM glucose suggests that NNT may impair insulin secretion via a diminished ATP/ADP ratio. There is evidence that NNT is involved in the detoxification of reactive oxygen species via the maintenance of a highly reduced glutathione pool through the NADPH-dependent glutathione reductase [36]. Indeed, ablation of NNT in *Caenorhabditis elegans* results in increased sensitivity to oxidative stress [37]. The *Nnt*KO glucose intolerant phenotype is proposed to be mediated via mitochondrial glutathione oxidation and elevated ROS leading to activation of UCP2, and the partial uncoupling of mitochondrial ADP phosphorylation from substrate oxidation [33], an idea also championed by others [38] (Fig. 1).

The aim of this study was to test the hypothesis that glucose intolerance in *Nnt*KO mice is mediated wholly or partly by UCP2. We looked for epistasis in double NNT and UCP2 knockout animals. If the hypothesis is correct, the effect of NNT knockout on glucose tolerance should disappear or decrease on a UCP2-null background.

## 2 Materials and methods

### 2.1 Generation of double NNT and UCP2 knockout mouse lines

Experiments were performed in accordance with regulations specified by the National Institutes of Health ‘Principles of Laboratory Animal Care, 1985 Revised Version’ and following U.K. Home Office ‘Guidelines for the Care and Use of Laboratory Animals’. Male and female mice were housed at 21 ± 2 °C and 57 ± 5% humidity in a 12 h light/12 h dark cycle, with standard laboratory chow and water ad libitum. *Ucp2^−/−^* (termed *Ucp2*KO) [21] mice (on a mixed 129/Sv and C57Bl/6 strain, therefore *Nnt^+/−^*) and *Nnt^−/−^* (termed *Nnt*KO) mice (pure C57Bl/6 strain) were crossed to generate mice heterozygous for each genotype (*Ucp2^+/−^*, *Nnt^+/−^*). Experiments using a mixed background strain may introduce noise into the data but the test for epistasis remains valid if the *Nnt*KO glucose intolerant phenotype is present. These heterozygous founders were then used to generate the four littermate genotypes (wild-type (WT), *Ucp2*KO, *Nnt*KO and *Ucp2*KO/*Nnt*KO) used in the following experiments at 3–5 months of age. Genotype was confirmed by PCR analysis and by western blotting of liver, kidney or pancreas mitochondria.

### 2.2 Genomic PCR and immunoblotting analysis

**Fig. 2:**
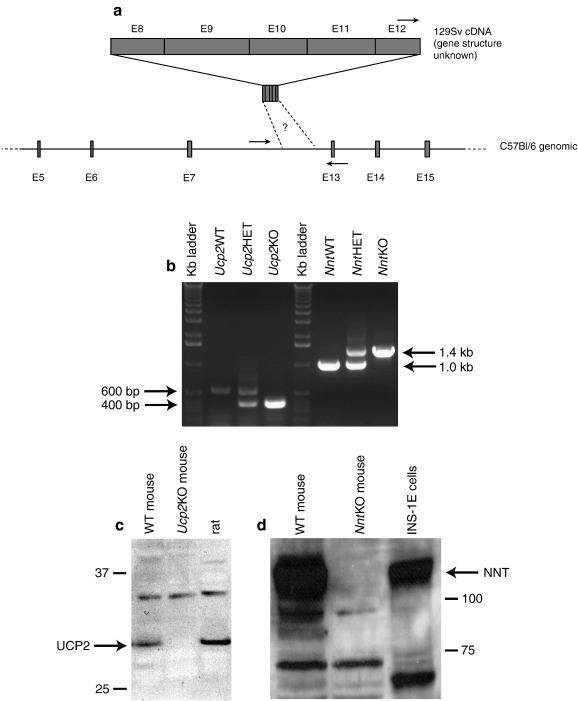
Generation of transgenic animals. Gene structure (exonic DNA — grey bars; non-coding DNA — black line) of the naturally occurring deletion mutation of the *Nnt* locus in C57Bl/6 mice is shown (a) and was assessed by PCR analysis (b) using the primers (arrows) indicated in (a). Ablation of the *Ucp2* gene locus according to [21] was also assessed by PCR using primers described therein (b). Western analyses of isolated mitochondria were used to detect UCP2 in pancreas (c) and NNT in liver (d).

PCR analysis of the *Ucp2*WT and *Ucp2*KO genomic locus was as described [21]. Primers were designed to detect the naturally occurring *Nnt* mutation found in some lines of C57Bl/6 mice as shown (Fig. 2b). The WT locus was identified using a sense primer (5′-ATGGGAGGGCATTTTTATCC-3′) in exon 12 and antisense primer (5′-CCAGAAAACCACCTTACCGA-3′) in intron 12. The *Nnt*KO locus was identified with a sense primer in intron 7, 5′ of the deletion event (5′-CTAAAACACATGCCCCGTCT-3′) and the antisense primer from intron 12. UCP2 and NNT proteins were detected as described in [27] and [33] respectively.

### 2.3 Glucose tolerance tests

Following overnight fasting, blood glucose levels were assessed using a glucometer (OneTouch, Lifescan, Milpitas, CA) prior to and then 10, 20, 30, 60, 120 and 180 min after i.p. injection of a bolus of glucose (1 g/kg). 13 male and 18 female, age matched, sibling paired mice were tested (total 31 mice) per group. Student's *t*-test was used to assess differences in blood glucose at given time points.

### 2.4 Metabolic phenotyping

Oxygen inhalation, carbon dioxide expiration and food and water intake were measured using an eight-chamber open-circuit oxygen-monitoring system controlled by CLAMS (Comprehensive Laboratory Animal Monitoring System; Columbus Instruments, http://www.colinst.com). Mice (sibling paired males aged 3–5 months) were housed individually in specially built plexiglass cages and were acclimatized for 72 h before data collection. Locomotor activity was simultaneously evaluated using an eight-cage rack OPTO-M3 Sensor system (Columbus Instruments) with measurements taken every 5 min throughout the dark and light cycles. ANOVA was used to test for differences between groups.

### 2.5 Proton leak kinetics

Proton leak rate across the mitochondrial inner membrane is directly proportional to the oxygen consumption rate measured in the presence of oligomycin to inhibit ATP synthesis [39]. The rate of proton leak is dependent upon its driving force, protonmotive force (measured as mitochondrial inner membrane potential in the presence of nigericin to convert pH gradients into electrical gradients). Oxygen consumption rate and membrane potential were measured simultaneously using electrodes sensitive to oxygen or the membrane-potential-dependent probe, triphenylmethylphosphonium (TPMP^+^). Mitochondria were isolated from the kidneys of 4 mice per group as described [39]. Proton leak kinetics were measured as described [11] with a TPMP^+^ binding correction assumed to be 0.35 (μl/mg protein)^− 1^. The time between energisation of mitochondria with succinate and titration of membrane potential with malonate was 1.5 min. Respiration rates (by linear interpolation between flanking points) were calculated at the highest membrane potential common to all conditions on a given day. ANOVA was used to test for differences in these respiration rates.

## 3 Results

### 3.1 Generation of a double NNT and UCP2 knockout model of glucose homeostasis

Mutation of the *Nnt* gene locus in C57Bl/6J mice was characterized by a 5 exon deletion (Fig. 2a) that leads to ablation of the protein as judged by western blot (Fig. 2d). *Ucp2*KO mice (a kind gift from Professor Bradford Lowell [21]) were crossed with C57Bl/6J (*Nnt*KO) mice to generate heterozygous founders. Heterozygote matings were then set up to generate sibling paired mice of the following genotypes of the double NNT and UCP2 knockout model; WT, *Ucp2*KO, *Nnt*KO and *Ucp2*KO/*Nnt*KO. The genotypes of individual mice were determined by PCR (Fig. 2b) and ablation of UCP2 or NNT protein was confirmed by western analysis (Fig. 2c and d).

### 3.2 Glucose homeostasis in the double NNT and UCP2 knockout model

**Fig. 3:**
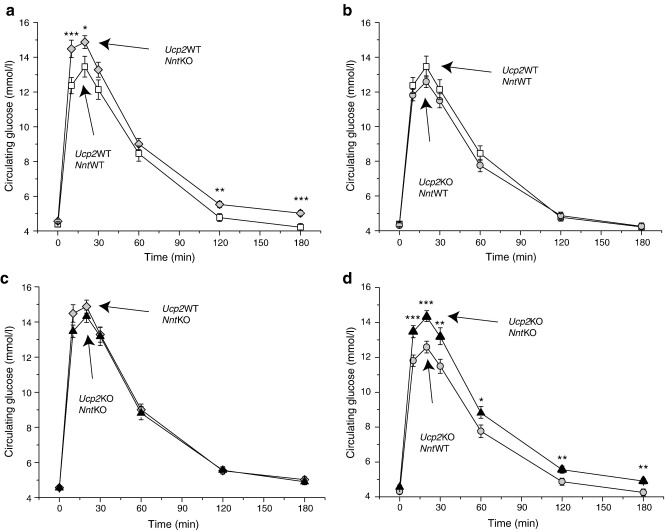
Glucose tolerance tests. I.p. glucose tolerance tests were performed in the double NNT and UCP2 knockout model following overnight fasting (a–d). These results are means ± SEM of experiments performed on *n* = 31 (13 male, 18 female) per group. Differences were assessed by unpaired Student's *t*-test: ⁎*p* < 0.05, ⁎⁎*p* < 0.02, ⁎⁎⁎*p* < 0.005.

The double NNT and UCP2 knockout model was used to assess the role of UCP2 in the glucose dysregulation phenotype seen upon ablation of *Nnt* [33]. Following overnight fasting, mice were injected i.p. with 1 g/kg glucose. Plasma glucose was then measured over a period of 3 h. As previously observed [33,35], ablation of *Nnt* alone led to a significant decrease in glucose uptake in the periphery when compared to WT mice (Fig. 3a), confirming the published effect of NNT ablation on glucose tolerance.

We were not able to replicate the improved glucose tolerance observed by Zhang et al. [21] upon ablation of UCP2 in an *Nnt*WT background (Fig. 3b). The improvement of glucose handling seen in *Ucp2*KO mice [21] was performed on a mixed C57Bl/6 × 129/Sv background. Although not all C57Bl/6 strains harbour the *Nnt* mutation, it is possible that the mice in [21] were at least heterozygous for mutation of *Nnt*. However, Fig. 3c shows that, in our hands, UCP2 ablation did not improve glucose tolerance in either *Nnt*WT or *Nnt*KO mice.

To test the dependence on UCP2 of the *Nnt*KO phenotype observed in Fig. 3a, the effect of NNT ablation on glucose tolerance was repeated in a *Ucp2*KO background (Fig. 3d). Our results show that NNT ablation significantly reduced glucose tolerance despite the absence of UCP2, showing that the presence of UCP2 is not required for the glucose intolerant phenotype to be observed in *Nnt*KO mice, that is, UCP2 is not a mediator of glucose homeostasis dysregulation in *Nnt*KO mice.

### 3.3 Energy balance in the double NNT and UCP2 knockout model

To assess whether there was any dysregulation of energy balance in the double NNT and UCP2 knockout model, metabolic parameters were assessed over a 72 h period (Table 1). No significant differences were seen in any of the parameters measured. However, ablation of *Nnt* alone tended to decrease locomotor activity (although this result did not reach statistical significance). Interestingly, locomotor activity was restored in the double knockout, hinting at a UCP2-mediated, *Nnt*KO locomotor phenotype. Further work is needed to follow up this intriguing finding.

### 3.4 Assessment of proton leak in kidney mitochondria isolated from the double NNT and UCP2 knockout model

**Fig. 4:**
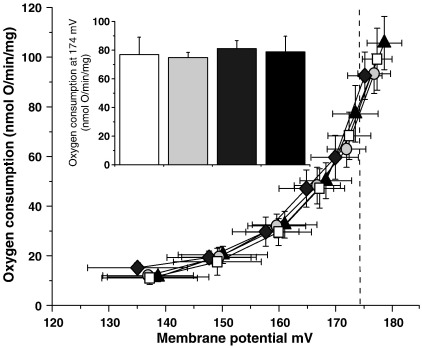
Proton leak kinetics were measured in mouse kidney mitochondria from WT (open square), *Ucp2*KO (light grey circle), *Nnt*KO (dark grey diamond) or *Ucp2*KO/*Nnt*KO (closed triangle) mice after 1.5 min incubation with substrate (4 mM succinate). The results are the means ± SEM of 7 experiments, each performed in duplicate. Respiration rates at a common driving force of 174 mV (inset) were not significantly different by ANOVA.

Kidney mitochondria were used as an abundant source of mitochondria containing both UCP2 and NNT to test whether ablation of *Nnt* led, as the hypothesis suggests, to a UCP2-dependent endogenous increase in mitochondrial proton conductance. To look for endogenous activation of UCP2, proton leak kinetics were measured in kidney mitochondria isolated from mice of the four genotypes generated in the double NNT and UCP2 knockout model (Fig. 4). After 1.5 min incubation with substrate, no significant difference in proton conductance was seen between the four genotypes tested. This suggests that, at least in isolated kidney mitochondria, absence of NNT does not promote an endogenous increase in proton leak either through UCP2 or through other pathways in the absence of UCP2.

## 4 Discussion

In this paper we describe the use of a double NNT and UCP2 knockout mouse model to examine the possible contribution of activation of mitochondrial proton leak through UCP2 to the poor peripheral glucose uptake phenotype seen in *Nnt*KO mice compared to their WT siblings. The principal findings of this paper are as follows. First, we were able to replicate the published observations [33,35] that ablation of *Nnt* alone leads to poorer glucose tolerance. Second, we were unable to replicate the published observation [21] that ablation of *Ucp2* alone leads to improved glucose tolerance. Third, we find that glucose tolerance is similar in *Nnt*KO mice whether or not UCP2 is present, showing that the *Nnt*KO glucose intolerance phenotype, at least in our experiments, is not mediated by UCP2. In addition, we find that the different genotypes show no significant differences in energy balance.

In mitochondria isolated from the kidneys of these mice, we find UCP2 expression (∼ 7 ng UCP2 per μg mitochondrial protein — data not shown) to be similar to that in rat kidney mitochondria [40]. We also find expression of NNT [41]. However, endogenous proton conductance is not different from WT in mitochondria from *Nnt*KO mice, as expected (N Parker, unpublished observations). Nor is it different from WT in kidney mitochondria from *Ucp2*KO mice, similar to the result obtained for endogenous proton conductance in skeletal muscle mitochondria from *Ucp3*KO mice [11]. Most importantly, endogenous proton conductance in mitochondria from double *Ucp2*KO/*Nnt*KO mice is not different from WT or from *Nnt*KO, showing that UCP2 is not endogenously activated in isolated kidney mitochondria under our incubation conditions when NNT is ablated.

We did not replicate the phenotype of improved glucose tolerance in *Ucp2*KO mice reported in [21], despite a large cohort (31 mice per group) and the clear presence of UCP2 in the WT pancreas (Fig. 2c). Loss of the *Ucp2*KO phenotype of improved peripheral glucose tolerance has also been seen by others when the *Ucp2*KO mutation is backcrossed away from a mixed 129/Sv × C57Bl/6 background [42]. However, acute UCP2 knockdown by RNAi in INS-1E insulinoma cells does alter mitochondrial coupling efficiency and insulin secretion [27], supporting the conclusions in [21]. This could suggest that either there are strain-dependent fluctuations in UCP2 function or could suggest that the chronic ablation of UCP2 triggers an unknown, strain-dependent compensatory mechanism. Similarly, while it was shown that ablation of NNT in a mixed strain (shown here) or a strain other than C57Bl/6 [33,35] caused a decrease in glucose tolerance, reciprocal correction of the *Nnt* gene locus in C57Bl/6 mice does not always lead to improvement in glucose tolerance [35]. Together, these disparate results may highlight the strain dependency and multigenic origin of the diabetic response, and suggest that the absence of UCP2 or presence of NNT can improve insulin secretion by pancreatic beta cells under some circumstances, but not under others, depending on factors yet to be identified.

Freeman et al. [33] showed that there was a difference in insulin secretion between WT and NNT knockdown in the pancreatic beta cell line, MIN6. This difference was normalised by tolbutamide, suggesting that action of NNT occurs prior to K_ATP_ channel closure. In *Nnt*KO pancreatic islets, ATP content was shown not to rise in response to elevated glucose, suggesting that the role of NNT in the regulation of insulin release is via a mechanism that affects ATP synthesis, directly or indirectly. While the uncoupling of mitochondrial substrate oxidation from ADP phosphorylation remains a candidate, increased proton leak into the mitochondrial matrix catalysed specifically by UCP2 does not seem to be the cause. It is noteworthy that mutations in other enzymes that lead to a decrease in the ATP/ADP ratio in beta cells result in a phenotype of reduced insulin secretion and impaired peripheral glucose uptake. For example, mutant forms of glucokinase [43,44], or inhibition of this enzyme [45] can lead to a severe diabetic phenotype, thought to be caused by changes in the ATP/ADP ratio. NNT catalyses the oxidation of NADH when mitochondrial membrane potential is high. It is possible that, in a finely tuned system (the pancreatic beta cell mitochondrion), increased levels of NADH per oxidised glucose molecule in the *Nnt*KO may inhibit the dehydrogenases of the tricarboxylic acid cycle, providing a negative feedback signal and reducing ATP synthesis. By casting doubt on the role of UCP2 in glucose dysregulation in NNT ablated animals, we open many other avenues to explore.

## Figures and Tables

**Table 1:** Whole animal measurements are not significantly different between genotypes.

	Weight (g)	Respiratory quotient (VCO_2_/VO_2_)	Energy expenditure (J/min/g)	Food intake (g/h)	Water intake (ml/h)	Ambulatory counts (counts/h)
WT	31.9 (1.1)	0.91 (0.01)	1.10 (0.03)	4.58 (0.35)	3.16 (0.35)	1517 (147)
*Ucp2*KO	32.0 (0.6)	0.91 (0.01)	1.10 (0.02)	4.63 (0.25)	2.52 (0.20)	1461 (124)
*Nnt*KO	33.0 (0.6)	0.92 (0.01)	1.07 (0.01)	5.52 (0.53)	2.57 (0.14)	1038 (52)
*Ucp2*KO/*Nnt*KO	32.2 (1.0)	0.91 (0.01)	1.09 (0.03)	5.38 (0.80)	2.60 (0.22)	1589 (232)

Values are means (± SEM) of *n* = 11/12 mice per group. ANOVA showed no significant difference between genotypes.
